# Tumor-associated M2 macrophages in the immune microenvironment influence the progression of renal clear cell carcinoma by regulating M2 macrophage-associated genes

**DOI:** 10.3389/fonc.2023.1157861

**Published:** 2023-06-08

**Authors:** Xiaoxu Zhang, Yang Sun, Yushuo Ma, Chengwen Gao, Youzhi Zhang, Xiaokun Yang, Xia Zhao, Wei Wang, Lisheng Wang

**Affiliations:** ^1^ Laboratory of Molecular Diagnosis and Regenerative Medicine, Medical Research Center, the Affiliated Hospital of Qingdao University, Qingdao, China; ^2^ Department of Lymphoma, the Affiliated Hospital of Qingdao University, Qingdao, China; ^3^ Department of Urology, the Affiliated Hospital of Qingdao University, Qingdao, China; ^4^ Department of Hematology, the Affiliated Hospital of Qingdao University, Qingdao, China

**Keywords:** M2 macrophages, renal clear cell carcinoma, tumor immune microenvironment, tumor-associated macrophages, M2 macrophage-related genes

## Abstract

**Background:**

Renal clear cell carcinoma (RCC) has negative prognosis and high mortality due to its early diagnosis difficulty and early metastasis. Although previous studies have confirmed the negative progression of RCC is closely related to M2 macrophages in tumor-associated macrophages (TAMs), the specific mechanism is still unknown

**Methods:**

We used immunofluorescence labeling and flow cytometry to detect the proportion of M2 macrophages in RCC tissues. And bioinformatics technique was used to obtain 9 M2 macrophage-related model genes, including *SLC40A1, VSIG4, FUCA1, LIPA, BCAT1, CRYBB1, F13A, TMEM144, COLEC12.* Using these genes, model formulas are constructed to devide samples into high and low risk groups, and then the overall survival (OS), progression-free survival (PFS) and Gene set enrichment analysis (GSEA) of the high and low risk groups were analyzed. Real-time quantitative polymerase chain reaction (RT-qPCR) was used to measure the expression of model genes between normal kidney tissue and RCC tissue, as well as between HK-2 cell and 786-O cell. Besides, we induced the M2 differentiation of THP-1 cell, and then co-cultured with the RCC cell 786-O in transwell to observe what effect M2 macrophages will cause on the invasion, migration and the expression of model genes of RCC.

**Results:**

Our study demonstrated M2 macrophages in RCC was about 2 folds that of normal renal tissue (P<0.0001) and M2 macrophages affected the prognosis of patients with RCC by affecting the co-expressed genes, which were mainly enriched in immune-related pathways. The results of *in vitro* experiments showed that in RCC tissues and 786-O cells, the model gene *FUCA1* was down-regulated, and *SLC40A1, VSIG4, CRYBB1* and *LIPA* were up-regulated. Besides, the results of co-culture showed that after 786-O co-culture with M2 macrophages, the ability of migration and invasion was promoted and the expressions of *FUCA1, SLC40A1, VSIG4, CRYBB1, LIPA* and *TMEM144* were all up-regulated.

**Conclusion:**

The proportion of tumor-associated M2 macrophages in RCC tissues is upregulated, and M2 macrophages promote the progression of RCC by regulating the expression of *SLC40A1, VSIG4, FUCA1, LIPA, BCAT1, CRYBB1, F13A, TMEM144, COLEC12* genes, thereby affecting the prognosis of patients with RCC.

## 1 Introduction

Renal clear cell carcinoma (RCC) is the most common pathological subtype of renal cancer (1, 2), originating from the proximal ureter (3). Due to the difficulties in early diagnosis, local invasion and early metastasis (4), about 30-40% of RCC patients occur metastasis at the diagnosis (5), which leads to poor prognosis and high mortality of RCC. Now due to the advancement of imaging technology, the detection rate of RCC has increased, which has improved the prognosis and reduced the mortality rate to a certain extent. Surgery serves as the most traditional treatment for RCC, but the metastasis or recurrence of the lesion limits this treatment. The emergence of new tyrosine kinase inhibitors (6)and targeted therapies such as PD-1/PD-L1 (7)has had a positive impact on the survival of metastatic renal cell carcinoma patients. However, the overall survival rate (OS) and progression-free survival (PFS) of RCC are still low (8), and the risk of metastasis and recurrence is still high (9). In recent years, increasing researchers have noticed the interaction between tumor cells and immune cells in the tumor immune microenvironment (TME) (10, 11), these immune cells are closely related to the occurrence and development of tumors (12), such as RCC (13). The components of the TME are very complex, including immune cells, stromal cells, extracellular matrix, and various secreted factors (14), among which macrophages account for a large proportion. Such macrophages infiltrating or accumulating in the microenvironment of solid tumors are defined as tumor-associated macrophages (TAMs), including M2 macrophages and a small part of M1 macrophages (15, 16). M1 macrophages are associated with tumor suppression, while M2 macrophages are associated with tumor promotion (17). Some studies have demonstrated that M2 macrophages in TAMs are significantly correlated with tumor stage, tumor cell differentiation, infiltration depth, angiogenesis, lymph node metastasis, and drug resistance, thereby affecting the prognosis of tumor patients (18), such as lung cancer, gastric cancer, and breast cancer (19). Previous studies have confirmed that M2 macrophages can promote the proliferation and migration of tumor cells by regulating certain genes in tumor cells, thereby affecting the prognosis of cancer patients, such as non-small cell lung cancer (20), squamous cell carcinoma of the lung (21) and lung adenocarcinoma (22), etc. This tumor genes regulated by M2 macrophages are called M2 macrophage-related genes. Previous studies have confirmed that M2 macrophages in the TME are closely related to negative prognosis of RCC (23, 24). However, so far, a comprehensive research of the biological role of tumor-associated M2 macrophages in the progression and clinical prognosis of RCC is still lacking. Therefore, in order to understand how M2 macrophages affect the prognosis of RCC, in this study, we used bioinformatics and *in vitro* experiments to explore the mechanism and screened nine M2 macrophage-related genes, including *SLC40A1, VSIG4, FUCA1, LIPA, BCAT1, CRYBB1, F13A, TMEM144, COLEC12*, and a new prognostic model was developed for RCC based on the nine M2 macrophage-associated genes. This model shows beneficial predictive effects. In addition, to investigate clinical and biological differences between high-risk and low-risk patients with RCC, clinical correlation analysis, enrichment analysis, and immune infiltration study were performed. In conclusion, we established a risk model based on M2 macrophage-associated genes for clinical prognosis prediction in patients with RCC. Else, it is necessary to identify and validate these predictive biomarkers as well as the prognostic model in the current treatment scenario of localized and advanced RCC in next researches, which makes the model we constructed in the study to assist the prognosis judgment of RCC and the evaluation of clinical treatment effect

## 2 Materials and methods

Materials: renal clear cell carcinoma cell line 786-O, normal renal cell line HK-2, THP-1 cells, fetal bovine serum (Procell, Wuhan, China), 1640 medium, DMEM medium (meilunbio, Dalian, China), 0.1% crystal violet dye, collagenase IV, hyaluronidase, DNA enzyme, Ficoll paque plus (Solarbio, Beijing, China), 8 μ m and 0.4 μ m transwell chambers, matrix glue (Corning, New York, USA). CD68, CD86 and CD206 flow antibodies, IL-4 and IL-10 (Biolegend, Beijing, China), primers (Sangon Biotech, Shanghai, China), PMA (MedChemExpress, New Jersey, USA), FIX&PERM Kit (MULTISCIENCES, Hangzhou, China). Reverse transcription Kit and qPCR Kit (TransGen Biotech, Beijing, China).

### 2.1 Data acquisition

The study used data from public databases. 537 samples of renal clear cell carcinoma were downloaded from TCGA database, including transcriptome data and clinical data. Among them, there were 613 cases in the transcriptional group (72 normal samples and 541 tumor samples). The survival time was between 0 days and 4537 days. GSE29609 is downloaded from GEO database and contains clinical information, such as sex, age, clinical stage, survival time, survival status and so on.

### 2.2 Acquisition of M2 macrophage related genes

We used CIBERSORT to calculate the proportion of 22 kinds of immune cell infiltration (TIC) in each sample of TCGA. According to the co-expression analysis of M2 macrophage expression and sample gene expression, M2 macrophage related genes were obtained, and the correlation coefficient between M2 macrophages and genes was obtained. Using Kyoto Encyclopedia of Genome and Genome (KEGG) (25) and Gene Ontology (GO) (26), the obtained genes were enriched and analyzed, and the biological process of M2 macrophage-related genes was determined.

### 2.3 M2 macrophage related genes were used to construct a prognostic model

The transcriptome data of TCGA and the data of GEO gene set were intersected and corrected, and the expression of M2 macrophage-related genes in each sample in the overlapping data was obtained by weighted gene co-expression network analysis (WGCNA). Then combined with the clinical information of TCGA and GEO respectively, univariate and multivariate Cox regression analysis was carried out to obtain meaningful prognosis-related genes. The obtained prognosis-related genes are intersected, which are the final prognosis-related genes. Based on the expression of prognostic related genes in TCGA, a lasso regression model was constructed and cross-validated to obtain the prognostic model formula, $\text{Riskscore}=\;\sum^{}\;\mathrm{Coef}\;*\;\mathrm{Exp}\;\left(\text{genes}\right)$ .

### 2.4 Survival analysis of patients with RCC using prognostic model

The prognostic model formula was used to score the TCGA samples, and the risk score was obtained. According to the median score, the samples were divided into two groups: high and low risk groups. The prognostic model formula was also used to score the GEO sample, and the median risk score of TCGA samples was used to divide them into high and low risk groups. Kaplan-Meier survival analysis was conducted to study the survival difference between high-risk and low-risk groups, and the model was verified by ROC curve to predict the accuracy of 1^st^, 3^rd^ and 5^th^ survival rates.

### 2.5 The way on which model genes work

GSEA was performed on the samples of high and low risk groups in order to find out the difference between the two groups. The difference of immune cell infiltration between high and low risk samples was analyzed, and the correlation between different immune cells and model genes was further analyzed. In addition, the gene expression related to the immune checkpoint was obtained from the sample gene, and the correlation with the risk score was analyzed.

### 2.6 Single gene analysis

The expression differences of individual model gene between normal tissue and tumor tissue and between tumor tissue and paracancerous tissue in the same sample were compared. In addition, through the expression of each gene in the prognostic model, the samples were divided into high and low risk groups, and then the survival differences between high and low risk groups were analyzed.

### 2.7 Verification by cell experiment

#### 2.7.1 Culture of HK-2 and 786-O cell

HK-2 was mixed with DMEM medium containing 10% fetal bovine serum and 1 × double antibiotic and seeded in petri dish. 786-O was mixed with 1640 medium containing 10% fetal bovine serum and 1 × double antibiotic and seeded in a petri dish. When the cells adhesion fusion degree reached 80%-90%, they were digested with 0.05% trypsin, subcultured, amplified and cryopreserved.

#### 2.7.2 Macrophages are extracted and detected surface markers

Renal cell carcinoma and normal kidney tissues were obtained from the affiliated Hospital of Qingdao University with the consent of the patients and their families. To remove the blood stain and necrotic tissues, the obtained tissues were washed with PBS containing 1 × double antibiotic, and then cut them into 1mm^3^ volume. The digestive juice was composed of 2mg/ml collagenase IV, 0.25mg/ml hyaluronidase, 0.2mg/ml DNA enzyme and 1640 medium. The tissues were digested at 37 °C for 2 hours. After digestion, the undigested tissue was removed with a 40 μm cell sieve. 5ml 1640 medium was used to re-suspended the cells, and then cell suspension was added to 5ml 100% Ficoll Paque Plus. The mixed solution was centrifuge at 1,500 r for 30 min. After centrifugation, the cells at the junction were taken. After washed once, the obtained cells were re-suspended with 1640 medium containing 20% FBS, and seeded on a 6-well plate. After 2 hours, unadherent cells were removed, and the adherent cells were labeled flow antibody CD68 and CD86. Besides, CD206 was labeled on the adherent cells after breaking the membrane. Last, macrophage markers were detected using flow cytometry.

#### 2.7.3 Effect of M2-type macrophages on the invasion and migration ability of 786-O

THP-1 cells were mixed and seeded on 6-well plate in 1640 medium containing 100ng/ml PMA to induce the cells maturation. 20ng/ml IL-4 and IL-10 were added to induce M2 differentiation. 48 hours later, the cells were digested with trypsin and labeled with flow antibodies CD68, CD86 and CD206 to detect whether the THP-1 cell successfully differentiated into M2 macrophages. The differentiated M2 macrophages were seeded to the 8 μm transwell chamber, and 786-O, which had been starved for 24 hours, was seeded into the upper chamber after the matrix glue was pretreated. 24 hours later, the 30 min was fixed with 4% paraformaldehyde, the fixed solution was removed. 10min was stained with 0.1% crystal violet, and then 5 visual fields were selected under the microscope after proper air-drying.

#### 2.7.4 Single gene analysis

The differences of individual model genes between normal tissues and tumor tissues of different samples, and of the same sample were compared. The survival differences between high and low risk groups of single model genes were analyzed. The results showed that *SLC40A1, VSIG4, FUCA1, LIPA, CRYBB1, TMEM144* 6 model genes among 9 model genes were different in 3 kinds of individual gene analysis (S1-S3). Therefore, we carried out *in vitro* qPCR of the 6 model genes. Trizol was used to extract the RNA of normal renal tissue and renal clear cell carcinoma, as well as HK-2 and 786-O. The differentiated M2 macrophages and 786-O cells were co-cultured in a 0.4 μm transwell chamber for 48 hours, then 786-O was digested and Trizol was added to extract RNA. The RNA extracted above were performed reverse transcription and qPCR (primer sequences in **Table 1**) to detect the expression of 6 model genes.

**Table 1:** Model gene primer sequences.

		Sequence (5’→3’)	Tm
*SLC40A1*	Forward Primer	CTACTTGGGGAGATCGGATGT	60.4
	Reverse Primer	CTGGGCCACTTTAAGTCTAGC	60.1
*VSIG4*	Forward Primer	GGGGCACCTAACAGTGGAC	62.0
	Reverse Primer	GTCTGAGCCACGTTGTACCAG	62.6
*FUCA1*	Forward Primer	GAAGCCAAGTTCGGGGTGTT	62.7
	Reverse Primer	GGGTAGTTGTCGCGCATGA	62.4
*LIPA*	Forward Primer	TCTGGACCCTGCATTCTGAG	61.0
	Reverse Primer	CACTAGGGAATCCCCAGTAAGAG	60.9
*CRYBB1*	Forward Primer	GTGCTCAAATCTGGCAGACC	61.0
	Reverse Primer	GGAAGTTGGACTGCTCAAAGG	60.8
*TMEM144*	Forward Primer	TATGGTTGGTTGCCTTGGTTG	60.8
	Reverse Primer	GTTCCCTGTTGCCCAAATGC	62.2

#### 2.7.5 Immunofluorescence labeling

The pathological sections were obtained from the Department of Pathology of the affiliated Hospital of Qingdao University. After dewaxing, hydration, antigen repair and inactivation of endogenous catalase, the sections were placed in PBS containing 3% BSA. The first antibody was incubated overnight at 4 °C. The second antibody was incubated at room temperature about 50min. After the sections were incubated and sealed, appropriate fluorescence channels were selected for observation and images were collected. Use image-J for data processing and analysis. The main antibodies are as follows: anti-CD68 and anti-CD163. All sections were evaluated by pathologists who did not know the identity and clinical outcome of the patient.

#### 2.7.6 Data analysis

The R4.2.1 software is used for statistical analysis, and the numerical value is $\bar{X}\pm \text{SD}$ . Kaplan-Meier curve and logarithmic rank test were used to evaluate the survival differences between groups. Univariate and multivariate Cox regression analysis were used to determine the prognostic factors. The correlation coefficient was calculated by Pearson and Spearman correlation analysis. Unmatched Student-t and Mann-Whitney U test were used to compare normal and abnormal variables, respectively. One-way analysis of variance and Kruskal-Wallis test were used as parametric and nonparametric methods to compare upper 2 groups. P< 0.05 was considered to be statistically significant.

## 3 Results

### 3.1 The clinical characteristics of the sample

The sample clinical data were obtained from TCGA and GEO, including the patient’s age, survival status, sex, grade and TNM stage (**Table 2**).

**Table 2:** The clinical characteristics of TCGA and GEO samples.

Clinical characteristics		TCGA (%)	GEO (%)
Total		n=537	n=39
Age at diagnosis		60.6 (26-90)	61.4 (21-78)
Survival status	Living	360(67.0)	23(60.0)
	Dead	177(33)	16(40.0)
Sex	Female	191(35.6)	15(38.5)
	Male	346(64.4)	24(61.5)
Histologic grade	G1	14(2.6)	1(2.5)
	G2	230(42.8)	12(30.8)
	G3	207(38.6)	11(28.2)
	G4	78(14.6)	15(38.5)
	GX	5(0.9)	–
	Unknow	3(0.5)	–
Stage	I	269(50.1)	12(30.8)
	II	57(10.6)	6(15.4)
	III	125(23.3)	19(48.7)
	IV	83(15.5)	2(5.1)
	Unknow	3(0.5)	–
T classification	T1	275(51.2)	11(28.2)
	T2	69(12.9)	5(12.8)
	T3	182(33.9)	22(56.5)
	T4	11(2.0)	1(2.5)
M classification	M0	426(79.4)	25(64.0)
	M1	79(14.7)	14(36.0)
	MX	30(5.6)	–
	Unknow	2(0.3)	–
N classification	N0	240(44.7)	31(79.5)
	N1	17(3.2)	5(12.8)
	NX	280(52.1)	3(7.7)

### 3.2 The role of M2 macrophages in RCC tissues

**Figure 1:**
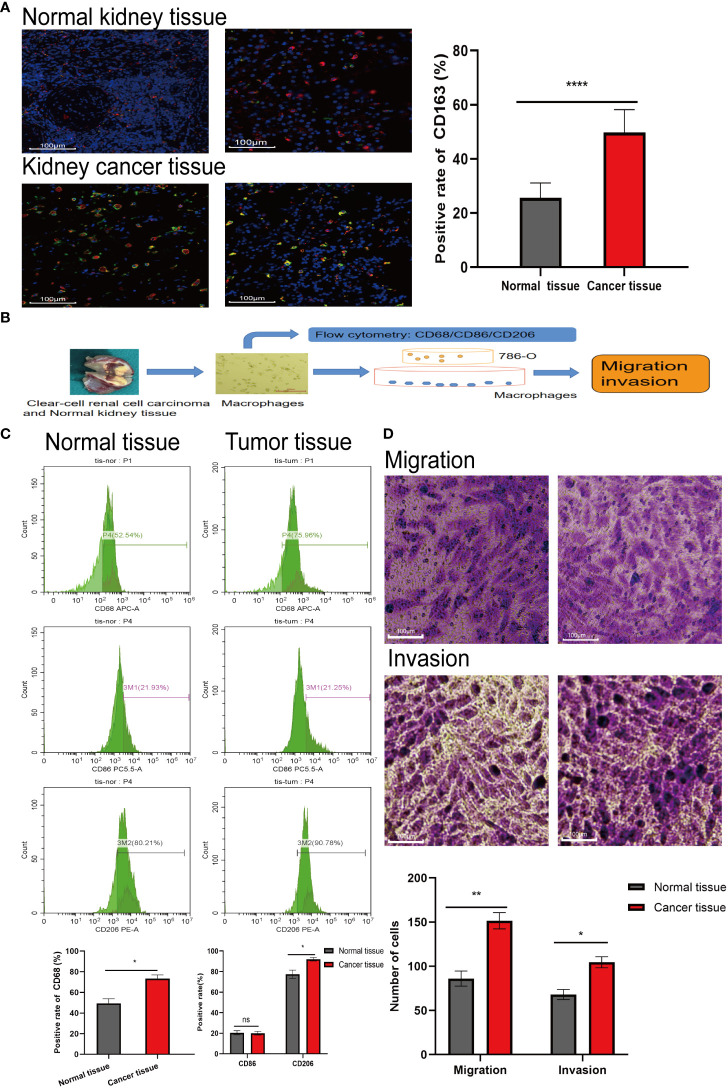
The proportion of M2 type macrophages elevates in RCC tissue. **(A)** Immunofluorescence labeling the normal kidney tissue and RCC tissue obtained, red fluorescence represents CD68 (labeling mature macrophages) and green fluorescence represents CD163 (labeling M2 type macrophages). **(B)** Extracting macrophages in kidney tissue and subsequent experimental procedure. **(C)** Macrophages were extracted from normal kidney tissue and RCC tissue, labeled with flow antibody CD68 (labeling mature macrophages), CD86 (labeling M1 type macrophages) and CD206 (labeling M2 type macrophages), and the proportion of M2 macrophages extracted from both tissues was detected by flow cytometry. **(D)** Macrophage cells extracted from the tissues were co-cultured with 786-O, and then crystalline violet staining to detect the effect of tissue-extracted macrophages on the invasion and migration ability of 786-O. The result of normal kidney tissue-extracted macrophages (left) co-cultured with 786-O. The result of RCC tissue-extracted macrophages (right) co-cultured with 786-O. *P<0.05, **P<0.01, ****P<0.0001.

The results of immunofluorescence labeling showed that the proportion of CD163 in RCC tissues was about 2 folds higher than that in normal renal tissues (P< 0.0001), which meant that the proportion of M2 macrophages in RCC tissues increased (**Figure 1A**). After M2 macrophages were extracted from RCC tissues and normal renal tissues according to the procedure (**Figure 1B**), the extracted cell markers CD68, CD86 and CD206 were detected by flow cytometry. The results confirmed that the proportion of M2 macrophages in RCC tissues was higher than that in normal renal tissues (P< 0.05) (**Figure 1C**). When the extracted macrophages were co-cultured with 786-O, the results showed that the macrophages extracted from RCC tissues could promote the invasion and migration of renal cancer cells more obviously (**Figure 1D**). This means that the enhancement of metastatic ability of RCC in the body is related to the increase of M2 macrophages.

### 3.3 Acquisition of M2 macrophage related genes

**Figure 2:**
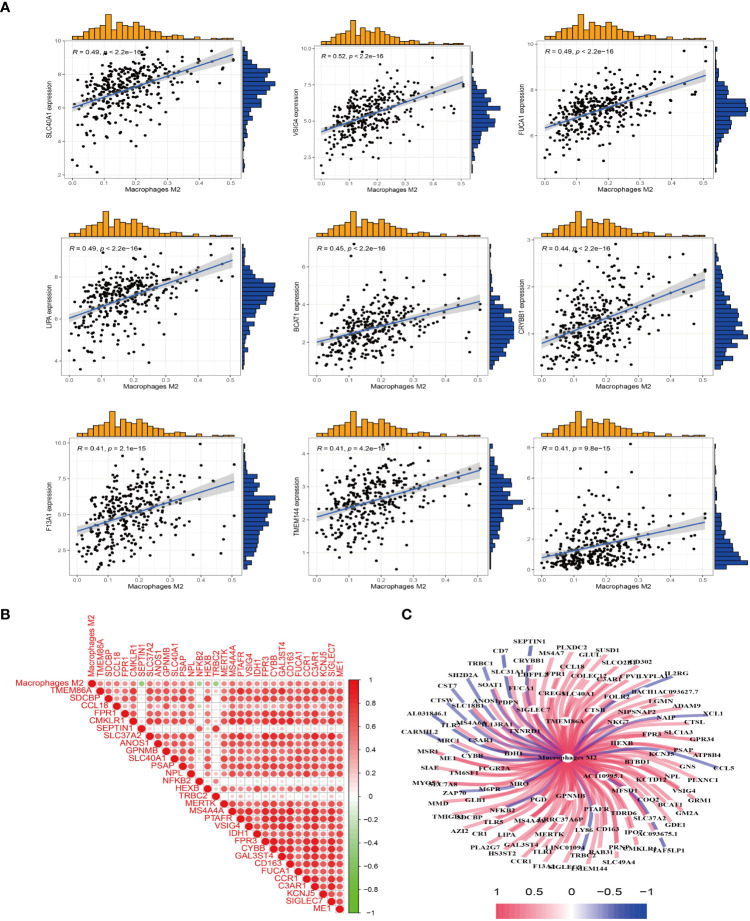
M2 macrophage-related genes were obtained. **(A)** Co-expression analysis between the sample genes and the expression of M2 macrophages was performed to obtain M2 macrophage-related genes. **(B)** Heat map of M2 macrophages co-expressed genes. Red represents positive correlation, and green represents negative correlation. **(C)** Visualization of M2 macrophage-related genes to obtain a co-expression network graph. Central node is M2 macrophages, connecting lines represent correlation, blue represents negative correlation, and red represent positive correlation.

After obtaining the proportion of immune cell infiltration in the TCGA sample, the correlation between the expression of M2 macrophages and the gene expression of the sample was analyzed. The results showed that there was a positive correlation between the expression of *SLC40A1, VSIG4, FUCA1, LIPA, BCAT1, CRYBB1, F13A, TMEM144, COLEC12* and M2 macrophages (**Figure 2A**). Furthermore, the results of coexpression analysis were visualized, and the correlation figure (**Figure 2B**) and coexpression network map (**Figure 2C**) were obtained. The visualized results allow visual observation of sample genes associated with M2 macrophages.

### 3.4 M2 macrophage related genes are mainly enriched in immunomodulatory pathway

**Figure 3:**
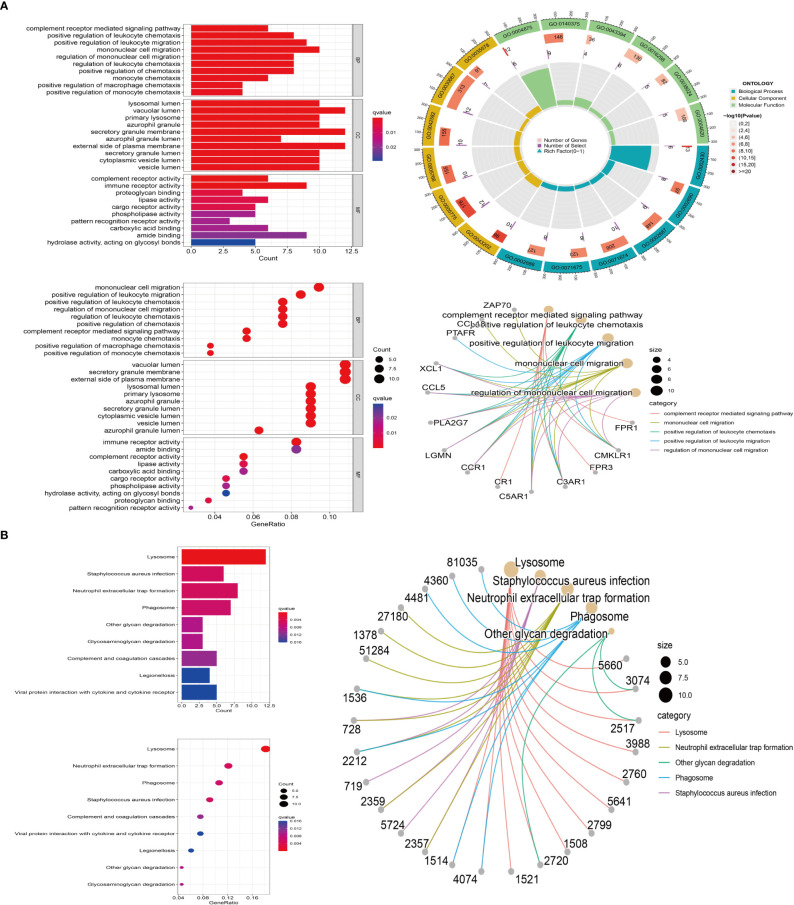
M2 macrophage-associated gene enrichment pathway. **(A)** GO enrichment. Red represents positive correlation, blue represents negative correlation (left); the outermost circle represents gene ID, and green, blue, yellow represent the three major functions, including molecular function, biological process, cellular component, respectively. The second circle represents the number of genes on each GO, the third circle represents the number of co-expressing genes enriched on each GO, and the fourth circle represents the proportion of co-expressed genes (top right); The major enrichment pathways of M2 macrophage-associated genes on GO (bottom right). **(B)** KEGG enrichment. Red represents positive correlation, blue represents negative correlation (left); The major enrichment pathway of M2-type macrophage-associated genes on KEGG (right).

Type M2 macrophage related genes were analyzed by GO and KEGG. GO enrichment results showed that M2 macrophage-related genes were mainly enriched in immune regulatory pathways, such as positive regulation of leukocyte, mononuclear cell migration, regulation of mononuclear cell migration, regulation of leukocyte chemotaxis (**Figure 3A**). KEGG enrichment results showed that M2 macrophage-related genes were also enriched in immune-related pathways, such as Neutrophil extracellular trap formation, phagosome (**Figure 3B**).

### 3.5 Construct a prognostic model for survival analysis

**Figure 4:**
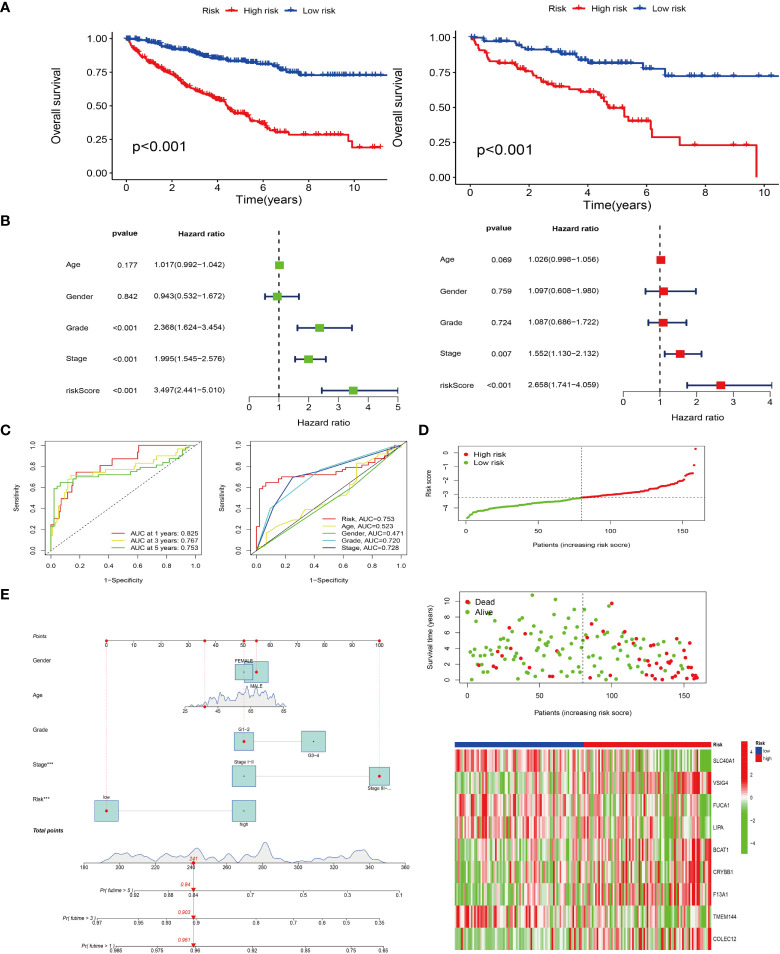
Prognostic models were constructed and survival analyses were performed. **(A)** Survival analyses for both high and low risk groups in the TCGA cohort (left); survival analyses for both high and low risk groups in the GEO cohort (right). **(B)** Single- and multi-factor independent prognostic analyses. Forest plots for single-factor Cox analysis (left); forest plots for multi-factor Cox analysis (right). **(C)** Risk models predicting patients’ survival at 1, 3, and 5 years by subject operating characteristic (ROC) curves (left); risk model combined ROC curves with other clinical traits (right). **(D)** Distribution of risk-score and survival status; Heat map of risk score and M2-type model genes. Red represents positive correlation and green represents negative correlation. **(E)** Column line plot of survival prediction model.

Based on the expression of prognosis-related genes in TCGA samples, a lasso regression model was constructed and cross-validated to get the model formula, risk score = VSIG4*0.235-SLC40A1*0.550-FUCA1*0.191-LIPA*0.066+BCAT1*0.138+CRYBB1*0.170+F13A1*0.138-TMEM144*0.090+COLEC12*0.048. Through the survival analysis of the prognosis model, the survival curves of TCGA and GEO samples were obtained respectively (**Figure 4A**). The results demonstrated that with the passage of time, the survival rate of both groups decreased gradually, but the overall survival rate of the high-risk group was significantly lower than that of the low risk group (P< 0.001), which means that our survival model is beneficial to divide the samples into high and low risk groups. We also conducted independent prognostic analysis of univariate and multivariate (**Figure 4B**). The results showed that our model could be independent of other factors as prognostic factors. In addition, judging the accuracy of our prognostic model in predicting patient survival by the ROC curve, we found that the prediction accuracy of our model is high. The AUC values of 1^st^, 3^rd^ and 5^th^ survival prediction are 0.825, 0.767, 0.753 respectively (**Figure 4C**). Moreover, the other ROC curve is built by combining our model with staging, grading and other clinical traits. Our model has the largest area under the curve (AUC=0.753), which means that our model is the most accurate in predicting patient survival (**Figure 4C**). In addition, the results of the risk curve (**Figure 4D**) show that the number of deaths increases as the patient’s risk increases. Using the line chart, the scores of all parturient traits were added to get a total score, and the survival time of patients was predicted according to the total score (**Figure 4E**). The probability of survival of more than 1 year, 3 years and 5 years was 0.961, 0.903 and 0.84, respectively.

### 3.6 Analysis of progression-free survival

**Figure 5:**
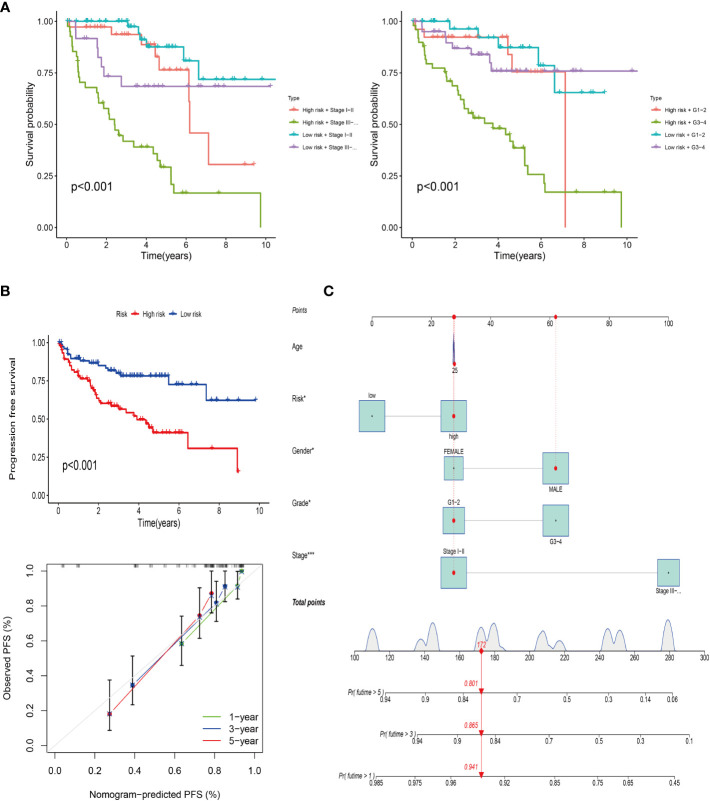
Progression-free survival of the risk model. **(A)** Survival analysis of the risk model combined with clinical stage (left) and grade (right). **(B)** PFS of the risk model and calibration curve. **(C)** Column line plot of the PFS prediction model.

The progression-free survival curve was obtained by combining the risk model with clinical traits such as staging and grading (**Figure 5A**). The results confirmed that with the passage of time, the overall survival rate of patients decreased, and the survival rate of high risk with late stage or grade sample was the lowest. PFS analysis was used to observe the progression-free survival time between the high and low risk groups. The results showed that the PFS time in the high-risk group was significantly lower than that in the low risk group (**Figure 5B**) (P< 0.001). The line chart predicted the probability that the PFS time of patients was more than 1 year, 3 years and 5 years, and the probability were 0.941, 0.865 and 0.801, respectively (**Figure 5C**).

### 3.7 The pathway of genes related to prognostic model

**Figure 6:**
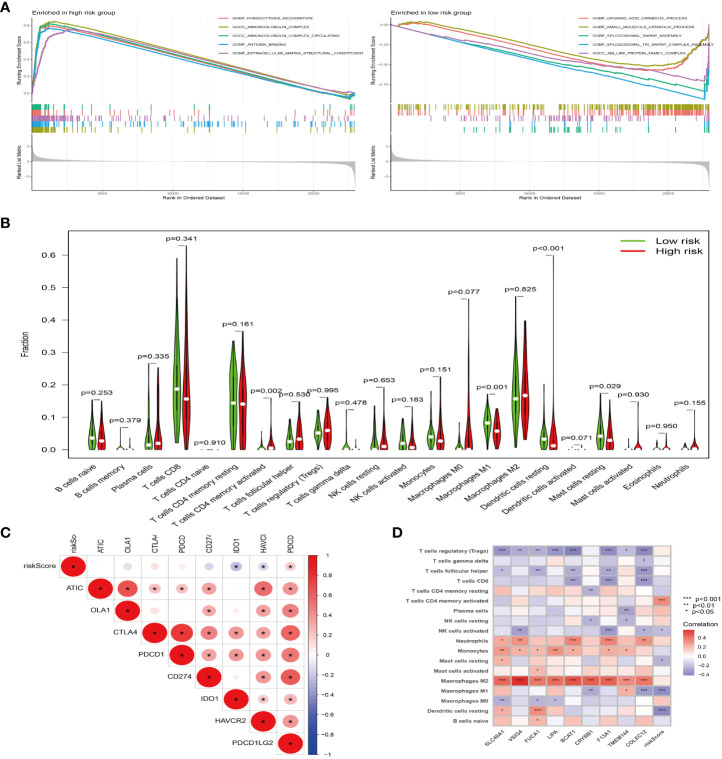
The model gene plays a tumor-promoting role through immunity. **(A)** GSEA of high (left) and low (right) risk groups, different color curves represent different functions or pathways. **(B)** Violin plot of immune cell difference analysis of high and low risk groups, green represents low risk group, and red represents high risk group. **(C)** Correlation analysis of immune checkpoint and risk score, “*” represents correlation, red represents positive, and blue represents negative. **(D)** Correlation analysis of immune cells and risk score, “*” represents correlation, red represents positive, and blue represents negative. *P<0.05, **P<0.01, ***P<0.001.

Through GSEA, we found that the prognostic model related genes in the high-risk group are active in immune-related pathways, such as Phagocytosis-receptor, immunoglobulin-complex, immunoglobulin-complex-circulating, and antigen-binding, while those in the low-risk group are mainly active in metabolic-related pathways, such as organic-catabolic-process and small-molecular-catabolic-process (**Figure 6A**). In addition, through the analysis of the difference of immune cells between high and low risk group, we confirmed that there are significant differences between high and low risk groups (**Figure 6B**). The results of correlation analysis between risk score and immune checkpoint (**Figure 6C**) showed that the risk score was correlated with *PDCDILG2, HAVCR2* and other immune checkpoint related genes. Besides, the results of immune cell correlation analysis (**Figure 6D**) showed that the risk score was related to T cell CD4 memory activated, macrophages M1 and other immune cells or immune pathways.

### 3.8 Effect of M2 macrophages on renal cancer cells

**Figure 7:**
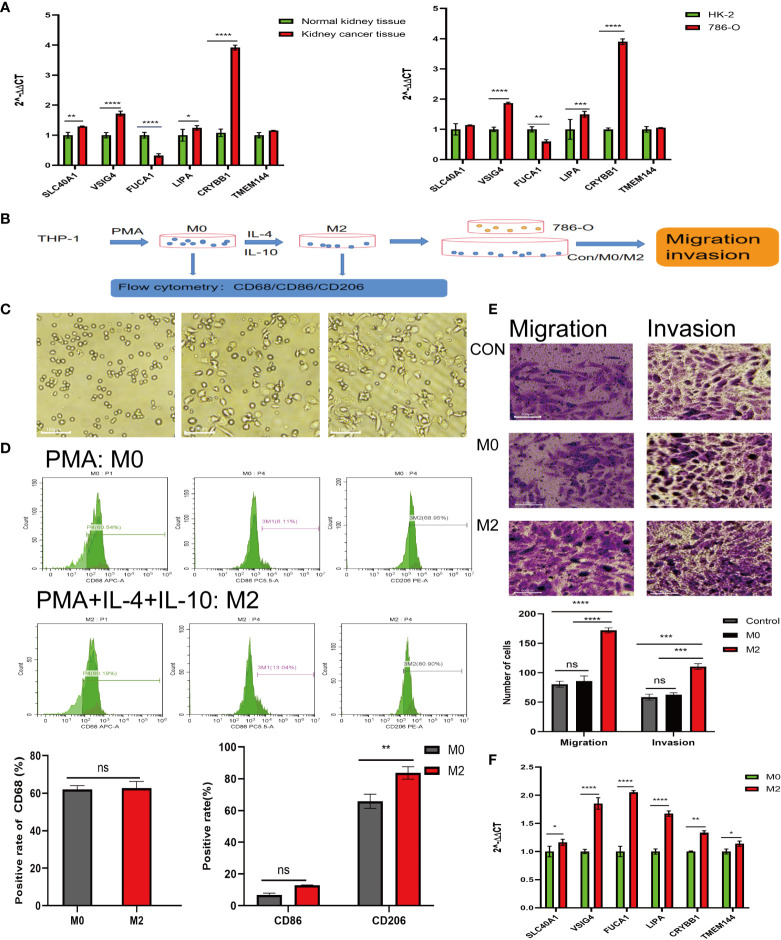
Experiments *in vitro* to detect the effect of M2-type macrophages on RCC. **(A)** Expression of model genes in normal kidney tissues, RCC tissues (left) and HK-2, 786-O (right). **(B)** Procedure of induced differentiation of M2-type macrophages from THP-1 and procedure of co-culture. **(C)** Undifferentiated THP-1 cells (left); mature M0 macrophages (middle); differentiated M2-type macrophages (right). **(D)** Expression of CD68, CD86 and CD206 after THP-1 differentiating. **(E)** Effect of M0 and M2 co-cultured with 786-O on migration and invasion ability of 786-O. **(F)** Expression of model genes in 786-O cells after co-culture. *P<0.05, **P<0.01, ***P<0.001, ****P<0.0001.

The high and low risk groups were constructed by each single gene in the model gene. The survival differences between the high and low risk groups were analyzed. And the results showed that there was a difference in survival between the high and low risk groups constructed by *VSIG4, TMEM144, SLC40A1, LIPA, F13A1* and *CRYBB1* (**Supplementary Figure 1**). In addition, the differences in the expression of M2 macrophage-related genes between tumor tissues and paracancerous tissues from the same sample were analyzed, and the results showed that there were differences in the expression of *VSIG4, TMEM144, SLC40A1, LIPA, CRYBB1, COLEC12, FUCA1* and *BCAT1* between tumor tissues and paracancerous tissues (**Supplementary Figure 2**). At the same time, the expression of M2 macrophage-related genes from normal tissues and tumors of different individuals was analyzed, and the results showed that there was a difference in *VSIG4, TMEM144, SLC40A1, LIPA, CRYBB1, COLEC12, FUCA1, BCAT1* between tumor tissues and normal tissues (**Supplementary Figure 3**). Based on the results of single gene analysis, 6 genes that cross existed in single gene analysis were selected from the 9 genes of the model gene. The differential expression of the model genes selected in RCC tissue and normal kidney tissue as well as in HK-2 and 786-O cells was analyzed. The results demonstrated that *FUCA1* was down-regulated in RCC tissue and cell, while *SLC40A1, VSIG4, CRYBB1* and *LIPA* were up-regulated in RCC tissue and cell (**Figure 7A**). In previous experiments, we confirmed that macrophages extracted from RCC tissues could significantly promote the invasion and migration of 786-O (**Figure 1D**). In further experiments (**Figure 7B**), in order to eliminate the effect of other cells that may exist in tissue extraction cells, we induced THP-1 cell to differentiate into M2 macrophages, and THP-1 were suspended cells under normal circumstances, while the M2 differentiation THP-1 were adherent cells (**Figure 7C**). Besides, the expression of flow antibodies CD206 were up-regulated after differentiating into M2 macrophages (**Figure 7D**). The results showed that M2 macrophages significantly promoted the migration (P<0.0001) and invasion (P<0.001) of RCC, compared with the control group and the M0 macrophages group (**Figure 7E**). After 786-O co-culturing with M2 macrophages, its RNA was extracted, and then qPCR was performed again to detect the expression of the above 6 model genes. The results showed that M2 macrophages promoted the expression of *VSIG4, TMEM144, SLC40A1, LIPA, FUCA1* and *CRYBB1* in 786-O cells, compared with the control group (Figure F).

## 4 Discussion

3Tumor immune escape, refractory and recurrence are attributed to tumor immune microenvironment to a large extent (27). Tumor-associated macrophages(TAMAs) play a key role in reshaping tumor immune microenvironment to promote tumor development (13). TAMAs are the most important component of immune cells in the tumor microenvironment of solid tumors (28). They can be roughly divided into two types: M1 of classical activation pathway and M2 of bypass activation pathway (29). Among them, M2 macrophages are mainly (27). Recently, studies have shown that tumor-associated M2 macrophages are associated with tumor staging, differentiation, metastasis and drug resistance, affecting the prognosis of tumor patients (30). Such as breast cancer, ovarian cancer, prostate cancer, cervical cancer, lung cancer and so on. However, few studies have focused on the effect of M2 macrophages on RCC.

Due to the difficulty of early diagnosis and early metastasis of RCC (4), 30% of RCC patients had metastatic lesions at diagnosis (5), which causes RCC poor prognosis and low survival rate. In our early study, immunofluorescence labeling was used to confirm the increase of M2 macrophages in RCC tissues. Besides, the macrophages extracted from renal clear cell carcinoma tissues and normal renal tissues were detected by flow cytometry, and the results also showed that the proportion of M2 macrophages in renal clear cell carcinoma tissues was significantly higher than that in normal renal tissues. In addition, the co-culture experiment confirmed that the macrophages extracted from RCC could promote the invasion and migration of renal cancer cells. Based on this, we speculate that the early metastasis and poor prognosis of RCC may be closely related to M2 macrophages in TAMs, but the specific mechanism of its effect is still unclear. Therefore, in this study, we used bioinformatics methods to explore the mechanism of M2 macrophages on RCC. Using the transcriptome data and clinical information of RCC downloaded from TCGA database and GEO database, we obtained M2 macrophage related genes in RCC, and then screened the model genes to construct a prognostic model. According to the prognosis model, it can be seen that the overall survival time and progression-free survival time of patients in the high-risk group are worse than those in the low-risk group, and, through univariate and multivariate analysis, it demonstrated that our survival model is not affected by age, sex, stage, grade and other factors, and can be used as an independent prognostic factor. The ROC curve confirmed that our prognostic model has high accuracy in predicting survival, which means that M2 macrophages in TAMs play a key role by affecting model genes, including *SLC40A1, VSIG4, FUCA1, LIPA, BCAT1, CRYBB1, F13A, TMEM144* and *COLEC12*, in tumor cells of patients with renal cell carcinoma. In addition, the results of GO and KEGG enrichment analysis showed that M2 macrophage-related genes were mainly enriched in immune-related pathways, such as positive regulation of leukocyte, mononuclear cell migration, regulation of mononuclear cell migration, regulation of leukocyte chemotaxis, neutrophil extracellular trap formation and phagosome. In addition, the results of GSEA showed that the prognostic model related genes in high-risk group were active in immune related pathways, such as Phagocytosis-receptor, immunoglobulin-complex, immunoglobulin-complex-circulating and antigen-binding, which means that the model genes affect the proliferation, invasion and metastasis of tumor cells through immune regulation, or M2 macrophages regulate the model genes of RCC through the formula of immune regulation. In addition, the correlation analysis between risk score and immune checkpoints as well as immune cells also showed that the model genes associated with M2 macrophages were closely related to immunity. Yet, although the link between risk scores and immune cells as well as immune checkpoints has been demonstrated in this study, there is no clinical evidence to support it, so prospective studies are necessary to validate the M2 macrophage-associated gene in the cohort of patients receiving immune checkpoint inhibitor (ICI) combination therapy in next studies. In addition, single gene analysis was carried out in this study, and *SLC40A1, VSIG4, FUCA1, LIPA, CRYBB1* and *TEME144* 6 genes were screened from 9 model genes. RNA was extracted from RCC tissue and normal kidney tissue, 786-O and HK-2 for qPCR. The results showed that *FUCA1* was down-regulated and *SLC40A1, VSIG4, CRYBB1* and *LIPA* were up-regulated in renal cell carcinoma and 786-O, and the difference was statistically significant. After 786-O co-culturing with M2 macrophages, RNA was extracted and qPCR was performed again. The results showed that compared with the control group, the expression of *FUCA1, SLC40A1, VSIG4, CRYBB1, LIPA* and *TMEM144* in M2 macrophage co-culture group were up-regulated. It has been previously reported that Fucosidase1 (*FUCA1*) encodes a lysosomal enzyme that participates in the degradation of fucose-containing glycoproteins and glycolipids. Down-regulation of *FUCA1* can enhance autophagy and inhibit macrophage infiltration, thus inhibiting tumor growth (31). In this study, M2 macrophages can up-regulate the expression of *FUCA1*, but its actual expression in renal clear cell carcinoma is down-regulated. We speculate that the contradictory result may be caused by the complexity of the tumor microenvironment, but the specific mechanism is still unknown. V-set and immunoglobulin domain containing 4 (*VSIG4*) is a transmembrane receptor of the immunoglobulin superfamily, which is specifically expressed in macrophages and mature dendritic cells. As a newly discovered B7 family-associated macrophage protein, it can inhibit T cell activation (32). Solute carrier family 40 member 1 (*SLC40A1*) is the only iron output protein found in mammals (33). It can increase the expression of iron in tumor microenvironment, and high iron levels can promote M2 polarization of macrophages (34). In addition, lysosomal acid lipase A (*LIPA*) contributes to the phenotypic transformation of macrophages and promotes the survival of macrophages (35), and studies have confirmed that *LIPA* deficiency can induce macrophages to differentiate into pro-inflammatory phenotype M1 (36–38).

In this study, we confirmed the increased proportion of M2 tumor-associated macrophages in RCC, which promotes RCC cell migration and invasion. The results of bioinformatics analysis show that M2 macrophages may play a significant part in promoting the development of renal cell carcinoma by regulating the expression of M2 macrophage related genes such as *SLC40A1, VSIG4, FUCA1, LIPA, BCAT1, CRYBB1, F13A, TMEM144* and *COLEC12* in RCC. Therefore, these genes may be considered as therapeutic targets in the treatment of renal cell carcinoma in the future, so as to provide more possibilities for the treatment of renal cell carcinoma.

## Data availability statement

The data used in this research are available from the corresponding author upon reasonable request.

## Ethics statement

The studies involving human participants were reviewed and approved by the Medical Ethics Committee of the Affiliated Hospital of Qingdao University (Approval No. QYFYWZLL27558). The patients/participants provided their written informed consent to participate in this study.

## Author contributions

XXZ, CG, XY and YZ conducted all the analysis. XXZ, YS and YM wrote the manuscript. XZ, WW and LW designed this work. All authors contributed to the article and approved the submitted version.

## Supplementary material

Supplementary Figure 1 Single model gene survival analysis. Red represents high-risk groups and blue represents low-risk groups.

Supplementary Figure 2 Differential analysis of individual model genes in normal and kidney cancer tissues from the same sample. Red represents normal samples and blue represents tumor samples.

Supplementary Figure 3 Differential analysis of individual model gene in normal kidney tissue and kidney cancer tissue from different samples. Red represents normal samples and blue represents tumor samples.
