# Supplementary figures and images for: Tumor-associated M2 macrophages in the immune microenvironment influence the progression of renal clear cell carcinoma by regulating M2 macrophage-associated genes
Source: Front Oncol. 2023 Jun 8;13:1157861. doi: 10.3389/fonc.2023.1157861 (PMC10285481; doi:10.3389/fonc.2023.1157861)

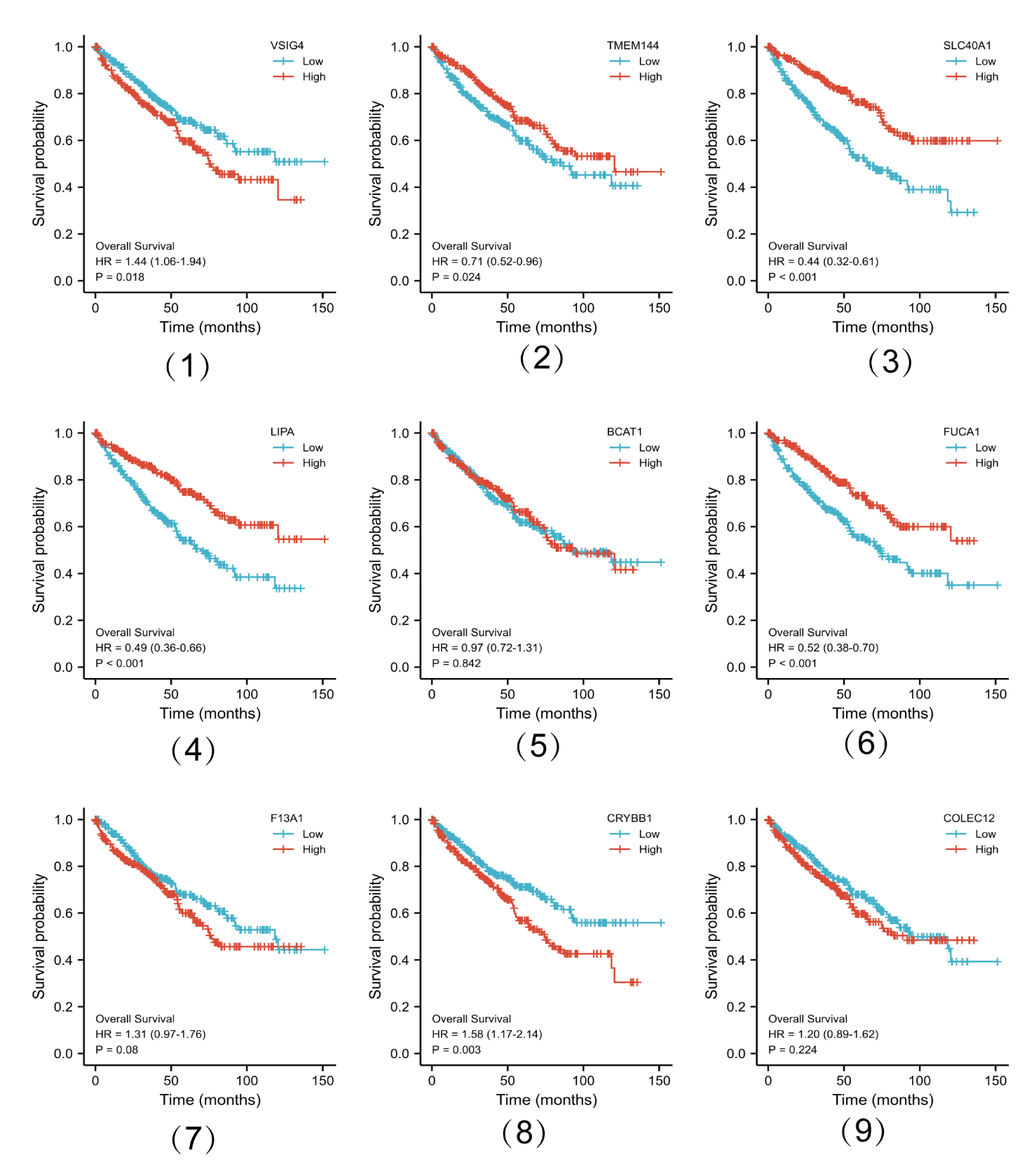

Supplement: Supplementary Figure 1 — Single model gene survival analysis. Red represents high-risk groups and blue represents low-risk groups. [file Image_1.tif]

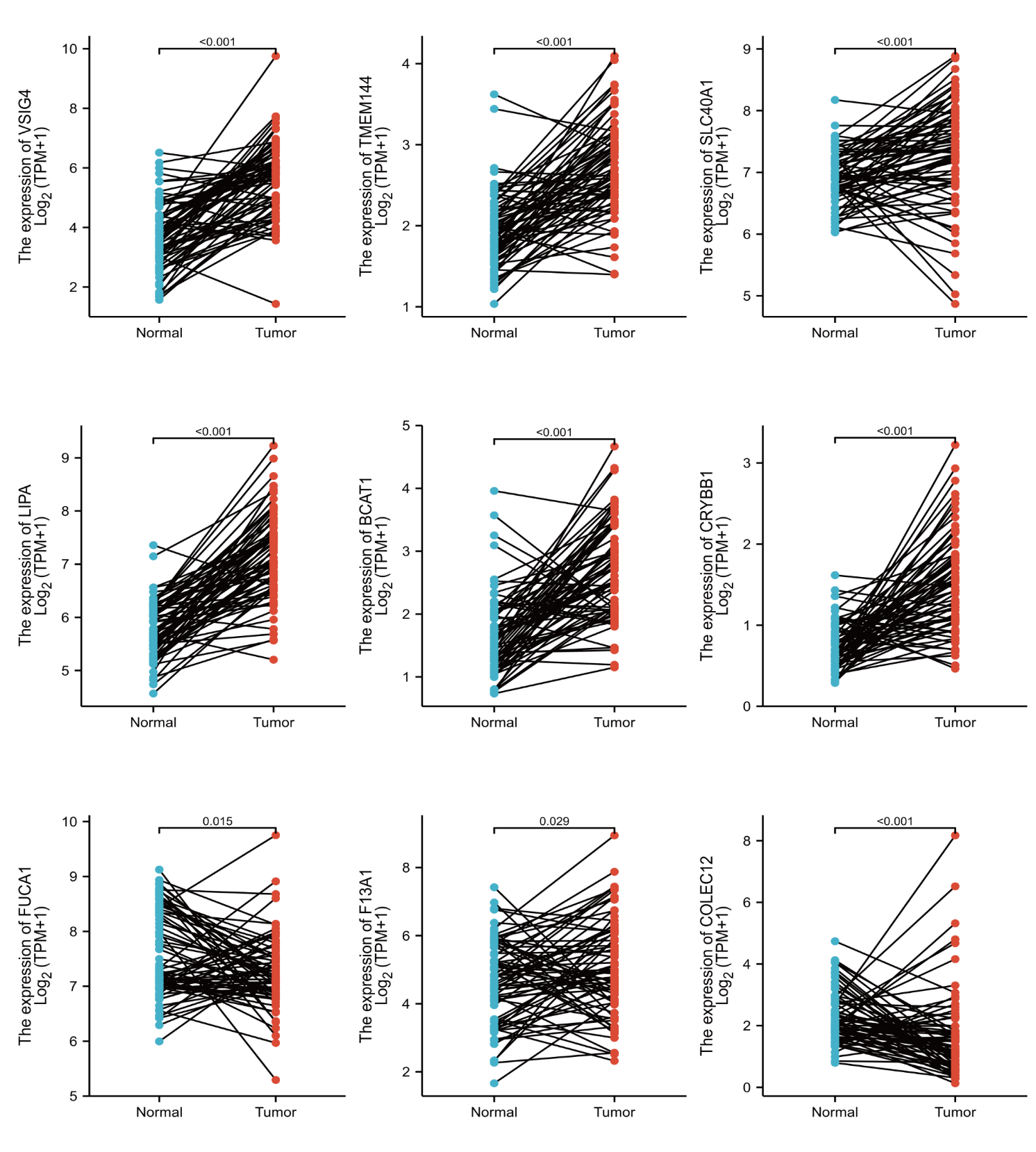

Supplement: Supplementary Figure 2 — Differential analysis of individual model genes in normal and kidney cancer tissues from the same sample. Red represents normal samples and blue represents tumor samples. [file Image_2.tif]

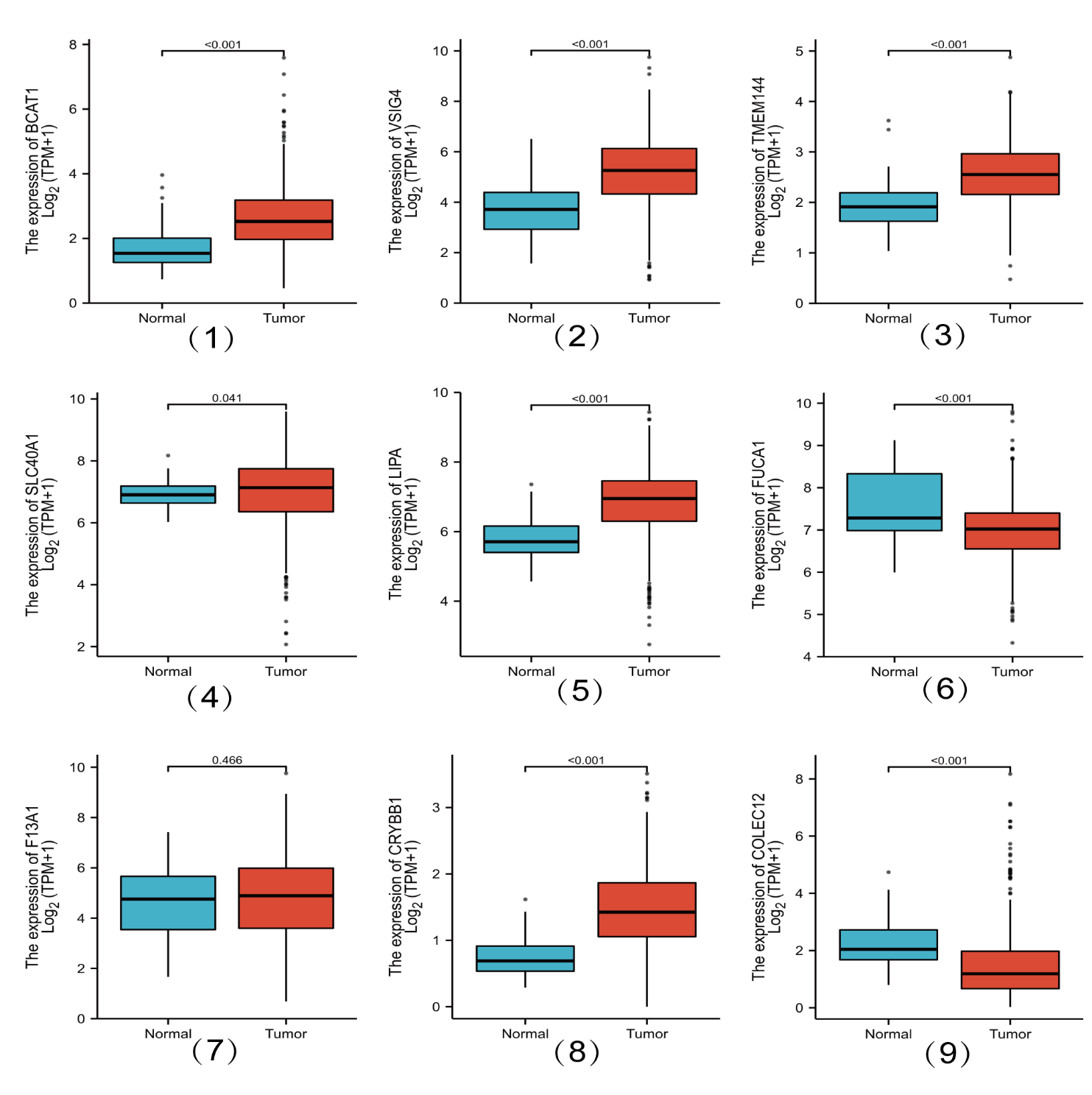

Supplement: Supplementary Figure 3 — Differential analysis of individual model gene in normal kidney tissue and kidney cancer tissue from different samples. Red represents normal samples and blue represents tumor samples. [file Image_3.tif]
